# Phenolic Melatonin-Related Compounds: Their Role as Chemical Protectors against Oxidative Stress

**DOI:** 10.3390/molecules21111442

**Published:** 2016-10-29

**Authors:** Annia Galano, Romina Castañeda-Arriaga, Adriana Pérez-González, Dun-Xian Tan, Russel J. Reiter

**Affiliations:** 1 Departamento de Química, Universidad Autónoma Metropolitana-Iztapalapa, San Rafael Atlixco 186, Col. Vicentina, Iztapalapa, 09340 Mexico City, Mexico; animor_ca@hotmail.com; 2 Consejo Nacional de Ciencia y Tecnología (CONACYT)—Departamento de Química, División de Ciencias Básicas e Ingeniería, Universidad Autónoma Metropolitana-Iztapalapa, Av. San Rafael Atlixco No. 186, Col. Vicentina, Iztapalapa, 09340 Mexico City, Mexico; adriana_perez_3@hotmail.com; 3 Department of Cellular and Structural Biology, UT Health Science Center, San Antonio, TX 78229, USA; Tan@uthscsa.edu (D.-X.T.); REITER@uthscsa.edu (R.J.R.)

**Keywords:** free radicals, scavenging activity, metal chelation, kinetics, reaction mechanisms, trends in activity

## Abstract

There is currently no doubt about the serious threat that oxidative stress (OS) poses to human health. Therefore, a crucial strategy to maintain a good health status is to identify molecules capable of offering protection against OS through chemical routes. Based on the known efficiency of the phenolic and melatonin (MLT) families of compounds as antioxidants, it is logical to assume that phenolic MLT-related compounds should be (at least) equally efficient. Unfortunately, they have been less investigated than phenols, MLT and its non-phenolic metabolites in this context. The evidence reviewed here strongly suggests that MLT phenolic derivatives can act as both primary and secondary antioxidants, exerting their protection through diverse chemical routes. They all seem to be better free radical scavengers than MLT and Trolox, while some of them also surpass ascorbic acid and resveratrol. However, there are still many aspects that deserve further investigations for this kind of compounds.

## 1. Introduction

Aerobic organisms are bound to the oxygen paradox, i.e., they cannot live without oxygen, but at the same time it represents a hazard to their health status [1]. The risk arises from the formation of oxidants, which is inherent to aerobic respiration. These species inflict structural damage to numerous molecules that are biologically important, including carbohydrates, lipids, proteins, and nucleic acids. Such damage is usually referred to as oxidative stress (OS), and can be potentiated by environmental and physiological factors that contribute to increase the oxidant amount. Some few examples are: pollution, radiation, consumption of certain drugs, cigarette smoke, heavy alcohol consumption, ischemia, infections, physical or mental stress, and aging [2,3,4,5,6,7,8,9,10,11,12,13,14,15].

OS has been held responsible—at least partially—for the onset and development of a wide spectrum of life threatening diseases like cancer [16,17,18], cardiovascular disorders [19,20,21], Parkinson’s and Alzheimer’s diseases [22,23,24,25,26,27], atherosclerosis [28,29,30], and diabetes [31,32,33]. Therefore, it is apparent that identifying molecules for protection against OS is a matter of vital importance. In addition to the enzymatic protection, there are many molecules that can offer chemical protection against OS. They are frequently referred to as antioxidants, and in the last decades have become the focus of numerous investigations.

**Figure 1:**
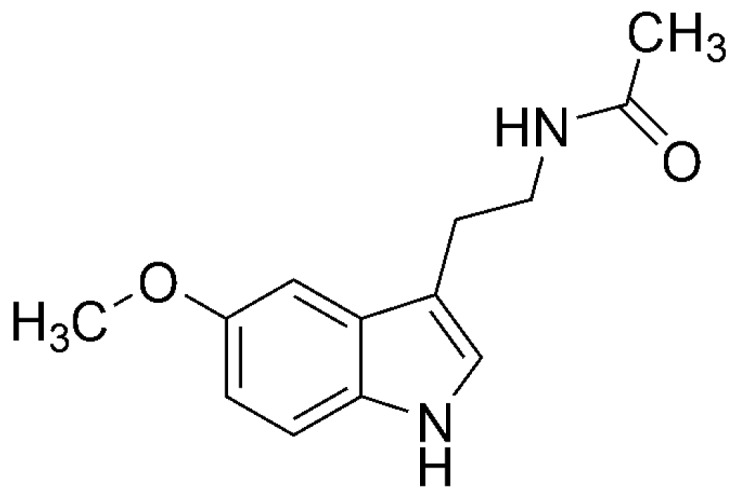
Structure of melatonin (MLT).

Among the molecules that offer chemical protection against OS, melatonin (MLT, Figure 1) and related compounds stand out [34,35,36]. There are several reasons why MLT has been proven to be particularly efficient for that purpose. It has very low toxicity, even at rather large doses [37]. It can easily cross physiologic barriers because of its optimal size, partial solubility in water and high solubility in lipids [38,39]. After been metabolized, MLT protection against OS does not decrease. In fact it is maintained, or even increased, due to the antioxidant capacity (AOC) of its metabolites [40,41,42,43,44,45]. Moreover, it has been proposed that this family of compounds can act in a “task-division” way, with some members of the family being particularly efficient as free radical scavengers, and others mainly behaving as metal chelators [43]. This way of action promotes a wide-ranging protection against oxidants.

On the other hand phenolic compounds have also been identified as efficient protectors against OS [46,47,48,49,50,51,52]. Therefore, it is not surprising that some phenolic melatonin derivatives are very efficient for that purpose [43,53]. In fact, they seem to be an appealing set of molecules in the battle against OS. As a result; this review focusses on the information gathered so far on these compounds. Different aspects relevant to their chemical protection against OS are reviewed, including location and functionality. A variety of reaction mechanisms involved in their AOC is analyzed. The data reported so far is used to propose trends in activity, based on comparisons with other antioxidants. In addition, some perspectives and current challenges regarding the role of MLT, and its phenolic related compounds, as protectors against OS are discussed.

## 2. Oxidative Stress and Free Radicals

More than half a century ago, in a pioneer work on the subject, Gerschman and coworkers [54] proposed for the first time that free radicals (FR) are the toxic intermediates associated with oxygen poisoning and ionizing radiation. The most representative feature of FR is that they have one, or more, unpaired electrons. This peculiarity makes them highly reactive and, consequently, very harmful species. FR are able of triggering chain reactions, propagating the molecular damage distant from the initial site of attack.

However, FR are not intrinsically dangerous. On the contrary, at low to moderate concentrations they have important physiological roles. For example FR are involved in the cellular signaling [4,6,9] and defense [7,8] systems, as well as in the maturation of cellular structures [2], mitogenic responses [3,4,5,6], regulation of insulin receptor kinase activity [7], and in the apoptosis of defective cells [55,56]. The toxicity of FR, and the resulting OS, arises as a consequence of a chemical imbalance between their production and consumption [57], which increases the FR concentrations above healthy levels. Under such conditions, free radicals can become a serious hazard to human health. For example, OS has been associated with neurological disorders [16,58,59,60,61,62,63,64], cancer [65,66,67,68,69], diabetes [70,71], pregnancy disorders, fetal defects and pre-eclampsia [72,73,74,75], as well as with cardiovascular [19,20,71,76,77,78,79], pulmonary [80,81,82], renal [20,83,84], and ocular [85,86,87] diseases.

There are abundant sources that contribute to increase the FR amounts in living organisms, thus promoting the deleterious effects of OS. Endogenously-produced FR arise from infection, inflammation, ischemia, immune responses, aging, and mental or physical stress [88,89,90,91,92,93,94,95,96,97,98]. On the other hand, cigarette smoke, environmental pollution, certain drugs, heavy or transition metals, alcohol, and radiation constitute exogenous sources that induce FR formation [99,100,101,102,103,104,105,106,107,108,109,110,111,112,113]. The most abundant FR, in vivo, are—or result from—reactive oxygen species (ROS), reactive nitrogen species (RNS), and reactive sulfur species (RSS), in that order. The names and acronyms of some of them are provided in Table 1.

The FR chemical reactivity is directly associated with the damage that they can inflict to biological molecules, with ^•^OH being the most reactive and dangerous species. It can react very fast (at, or close to, diffusion-controlled rates) with a large variety of molecules [114]. Thus, ^•^OH can damage almost any chemical species in the vicinity of its formation site [115]. This radical is responsible for most of the ionizing radiation-induced tissue damage [116] and easily oxidizes DNA [117,118,119]. RO^•^ are less reactive than ^•^OH and more reactive than ROO^•^, as long as R is the same—or similar—in both species [120,121,122,123,124]. In the context of OS, ROO^•^ species are particularly important. They are capable of diffusing to remote cellular locations, and are among the main products yielded by lipid peroxidation [125]. ROO^•^ are also among the FR that can be successfully scavenged to retard OS [126] because their half-lives are not too short, which allows antioxidants to efficiently deactivate them before they harm biological targets [127].

The hydroperoxyl radical (HOO^•^) is the smallest of the ROO^•^, and the conjugated acid of O_2_^•−^. The acid-base equilibria involving these two oxidants is crucial to OS since most of the damage attributed to the HOO^•^/O_2_^•−^ pair is actually inflicted by HOO^•^, albeit it represents only about 0.3% of the HOO^•^/O_2_^•−^ pair in the cytosol of a typical cell [128]. It has been demonstrated that the reactions of unsaturated fatty acids with HOO^•^ are ~5 orders of magnitude faster than with O_2_^•−^ [129], and that HOO^•^ is actually responsible for initiating fatty acid peroxidation [130]. In addition, HOO^•^ is more reactive than other ROO^•^ species when R is an alkyl or an alkenyl group [120,131], while this trend is reversed when R is a group with higher electron-withdrawing character (for example R = −CCl_3_) [131,132].

Regarding RNS, the direct toxicity of ^•^NO is expected to be minor due to its rather low chemical reactivity [133,134]. However, when reacting with O_2_^•−^, ^•^NO can produce a significant harmful species (ONOO^−^) [135], which is capable of chemically damaging lipids, proteins and DNA [136,137,138], The reactivity of ^•^NO_2_ is in between those of ^•^NO and ONOO^−^, which makes it a moderate oxidant [139]. On the other hand, RSS are yielded by the reactions of thiols with ROS and RNS [140], thus RSS are expected to be less reactive than the corresponding O and N species. However, this does not mean that they are not dangerous. On the contrary, it has been demonstrated that RSS are capable of damaging proteins [141,142,143].

## 3. Chemical Antioxidants

Chemical antioxidants are species that offer protection against OS by non-enzymatic ways of action, and have been proven to be helpful in the prevention and treatment of numerous OS-related health disorders [10,144,145,146,147,148,149,150,151]. They can be endogenously produced or acquired from foods, or diet supplements. Some examples of endogenous chemical antioxidants are melatonin, coenzyme Q10, glutathione, and lipoic acids; albeit some of them can also be involved in the enzymatic defense system. Exogenous chemical antioxidants come from numerous sources, which can be classified as natural or synthetic depending on how they are produced. Phenolic compounds, carotenes, tocopherols and ascorbic acid are a few examples of natural exogenous antioxidants. On the other hand, *N*-acetylcysteine, gallates and edaravone, are synthetically produced chemicals with important antioxidant activity.

### 3.1. Mechanisms of Action

Chemical antioxidants are diverse not only in their sources and structures, but also in the reaction mechanisms contributing the most to their protection against OS. This particular feature allows to differentiate between primary (chain breaking or Type I) and secondary (preventive or Type II) antioxidants [152]. Primary antioxidants prevent oxidation by scavenging FR, i.e., by directly reacting with them, yielding significantly less reactive species or terminating the radical chain reaction. On the contrary, secondary antioxidants retard oxidation by indirect means of action, i.e., by chemical routes that do not involve direct reactions with FR. For example, by metal chelation, decomposition of hydroperoxide into non-radical species, repairing primary antioxidants, deactivating singlet oxygen, or absorbing ultraviolet radiation. Some species, however, can behave as multiple-function antioxidants, i.e., exhibiting both primary and secondary AOC.

The most common reaction mechanisms involved in the primary AOC exerted by chemical antioxidants are schematically represented in Table 2. The RAF route involves the addition of the free radical to the antioxidant. Thus, the most important structural feature of the later is the presence of multiple bonds. Other important aspect regarding this route is that it may be limited by steric effects, and the radical’s electrophilicity. The more electrophilic the radical, the more likely it is involved in RAF reactions. On the other hand, SET reactions can occur through two different routes, depending on which species is the electron acceptor: the radical (SET-I) or the antioxidant (SET-II), with SET-I being common one.

Since PCET and HAT reactions yield exactly the same products, it is not easy to differentiate between them. In fact, when a global chemical process consists on an H transfer it is frequently assumed to be a HAT reaction, albeit it might actually correspond to PCET. In HAT reactions the proton and the electron are transferred together as a single entity, i.e., a hydrogen atom. On the contrary, in PCET reactions the electron and proton are transferred as two separated particles, but in a single step, without any stable intermediate. A common way of describing PCET is as a reaction with a proton and electron transferred from different sets of orbitals. Thus, while in HAT the donor and the acceptor are the same for both particles (the electron and the proton), in PCET they are different.

The SPLET and SEPT mechanisms both involve two elementary steps: one proton transfer and one electron transfer, but in the opposite order. The SPLET mechanism was first proposed by Litwinienko and Ingold [153] for the reactions of phenolic compounds. Currently, there is an overwhelming amount of evidence supporting the crucial role of this mechanism on the AOC of this family of compounds. The SEPT mechanisms is less common, albeit it is involved not only in the free radical scavenging processes but also in the oxidation of biological targets, such as the nucleosides in DNA [119].

The information regarding the reaction mechanisms involved in secondary AOC is less abundant than that already gathered for primary AOC. Metal chelation is particularly appealing when it inhibits the ^•^OH production, which—as mentioned before—is one of the most damaging oxidants in biological systems. Within the cells, ^•^OH is mainly produced by the Fenton reaction or the Haber-Weiss recombination (HWR). The latter can be globally written as:

O_2_^•−^ + H_2_O_2_ → ^3^O_2_ + OH^−^ + ^•^OH

However, it only becomes physiologically important when it is catalyzed by metal ions [154], which actually corresponds to a two-steps reaction:

M^q+^ + O_2_^•^^−^ → M^(q−1)^ + ^3^O_2_

M^(q−1)^ + H_2_O_2_ → M^q+^ + OH^−^ + ^•^OH

The second step is the so-called Fenton reaction. The most likely metals involved in these reactions are iron and copper, which in biological media are mainly found as Fe(III) and Cu(II). Accordingly, the first step of the HWR is expected to play a crucial role in the ^•^OH production. That is why chelating agents able to inhibit the reduction of Fe(III) and Cu(II) may be effective in downgrading, or inhibiting, the ^•^OH production and the associated OS. In fact, metal chelation has been proposed as a therapy for Alzheimer’s disease [155].

There is more than one reaction mechanism that might be involved in metal chelation. The most frequently assumed is the direct chelation mechanism (DCM):

M^q+^ + H*_n_*Atx → M(H*_n_*Atx)^q+^

While the coupled deprotonation-chelation mechanism (CDCM) might arise when the antioxidant has acid-base equilibria [156]:

M^q+^ + H*_n_*Atx → M(H*_n_*_−*m*_Atx)^q−*m*^ + *m*H^+^

Metal chelation is also a crucial step in the ^•^OH-inactivating ligand (OIL) [157,158] behavior of chemical species, which can protect against ^•^OH-induced damage in two different ways [159]: (i) sequestering metal ions from reductants by inhibiting the reduction of metal ions; or (ii) deactivating ^•^OH after being produced via Fenton-like reactions. In the latter ^•^OH are still produced, but they are immediately scavenged by the OIL acting as a ligand in the metal chelate.

Secondary AOC by single oxygen quenching (SOQ) may involve different routes, including:

^1^O_2_ + Atx → ^3^O_2_ + Atx

^1^O_2_ + Atx → AtxO_2_

The first one is a singlet oxygen physical quenching, with the antioxidant remaining chemically unchanged; while the second actually corresponds to a singlet oxygen chemical quenching, i.e., a new chemical species is yielded. The physiological importance of antioxidants able to quench ^1^O_2_ has been related, for example, to the protection of the skin from exposure to ultraviolet-A radiation [160]. Secondary antioxidants can also exert their protection by repairing primary antioxidants and biomolecules. These repair processes usually involve hydrogen or electron transfer reactions.

#### Main Reaction Mechanisms Involved in AOC Assays

Currently there are numerous experimental assays that can be used to measure the antioxidant capacity (AOC) of chemical compounds. However, the main reaction mechanism governing the outcome of different AOC assays may not be the same. Therefore, it is important to know what the dominant chemical route is for each of them. This knowledge is crucial to select the most appropriate method, depending on the antioxidant to be tested, and also to properly interpret the obtained results. Some of the most widely used AOC assays are briefly summarized here, emphasizing on the features most relevant to the context of this review. The interested reader can find more detailed information on these—and other—AOC assays elsewhere [161,162,163,164,165,166].

*Oxygen radical absorbance capacity* *(ORAC) assay*: It measures the radical chain breaking ability of antioxidants by monitoring the inhibition of peroxyl radical-induced oxidation. This technique usually involves using Trolox as the antioxidant reference, and—as a result—the AOC of the tested antioxidants is reported as Trolox equivalents. ORAC essentially measures the hydrogen atom donating ability of antioxidants, thus it can be considered as an HAT-based method. This assay was originally developed for measuring hydrophilic antioxidants, but later it has adapted for both lipophilic and hydrophilic species [167].

*2,2-Diphenyl-1-picrylhydrazyl* *(DPPH) assay*: It is based on the neutralization of the DPPH radicals by antioxidants mainly by electron transfer, which implies that AOC is ruled by the electron donating capacity of the tested molecules. However, it has been suggested that the HAT route might also be involved as a marginal pathway [162,168]. Albeit the DPPH assay is among the most frequently used to obtain a first AOC evaluation, it has been argued that the results obtained with this method are not necessarily extrapolable to biological systems [169]. Moreover, its applicability for ranking antioxidants has been recently questioned [170].

*Trolox equivalent antioxidant capacity (TEAC) assay*: It measures AOC as the ability of antioxidants to scavenge the radical cation known as ABTS^•+^ (2,2′-azinobis(3-ethylbenzothiazoline-6-sulphonic acid)). Although it is usually classified as an ET-based method, the HAT mechanism can also contribute to the observed AOC. The relative importance of these two chemical routes is mostly determined by the structure of the antioxidant and the pH of the medium [162]. TEAC can be conducted in both hydrophilic and lipophilic media and is not significantly affected by the ionic strength of the medium [171]. The results obtained by this assay are frequently reported as Trolox equivalents.

*Ferric reducing antioxidant power (FRAP) assay*: It measures AOC as the capability of antioxidants to reduce Fe(III) or Fe(III)-ligand complexes, in acidic media. Thus, it is a typical electron transfer-based method. Therefore, it has been argued that it cannot be related with HAT-based AOC occurring in lipid systems but it can be used—in combination with other methods—for identifying the main reaction mechanisms involved in AOC [162]. Albeit the conventional FRAP assay was designed to test hydrophilic antioxidants, it was modified to enable simultaneous measuring the AOC of both hydrophilic and lipophilic molecules [172].

Theoretical chemistry can also be used to estimate AOC. A computational protocol has been recently developed to produce reliable quantitative data concerning the kinetics of radical-molecule reactions in solution. It is commonly referred to as the quantum mechanics-based test for overall free radical scavenging activity (QM-ORSA) [173], and has been extensively used to produce trends in primary AOC. There are some key points when using this protocol. It is required to use the same FR, and solvents of similar polarity, to assure the fairness in the comparisons; and calculation methods that are reliable for kinetics, such as LC-ωPBE, M06-2X, BMK, B2PLYP and M05-2X [174]. The QM-ORSA protocol has been validated by comparison with experimental results, and its uncertainties have been proven to be no larger than those arising from experiments [173]. It allows the use of two different scales: (i) the absolute, based on overall rate coefficients; and (ii) the relative, using Trolox as reference, and making separately analyses in aqueous and non-polar (lipid-like) media. In addition, it accounts for a wide variety of reaction mechanisms including SET, HAT, SPLET and RAF.

### 3.2. Melatonin (MLT)

MLT is an indoleamine with two side chains, a 5-methoxy group and 3-amide group. Its molecular weight is 232.2 g/mol, it has 17 heavy atoms and log*P* = 1.4. Accordingly, the MLT size, partial solubility in water and high solubility in lipids, promotes that MLT can easily cross physiologic barriers. Other relevant physicochemical properties of MLT are the number of hydrogen bond acceptors (4), bond donors (2), and rotatable bonds (4) as well as its molar refractivity (65.6) [53], topological polar surface area (54.12 Å^2^) [53] and pKa (12.3) [175]. According to the latter, it is expected that at physiological pH (7.4) neutral MLT is by far the dominant acid-base form of this compound.

On the other hand, MLT reduction potential has been reported to be 0.95 V [176], which indicates that it may interact with the respiratory complexes of the electron transport chain by donating and/or accepting electrons. This behavior is expected to increase electron flow, which is an effect that not all other antioxidants possess [177,178]. MLT exhibits an anodic wave at a maximum potential value of 0.73 V but not wave in the reverse scan [179], which was attributed to the possible instability of the oxidation products as a consequence of fast second order decay of the radicals. In addition, the presence of the anodic wave constitutes evidence of the electron donating ability of MLT and supports the electron donation hypothesis used to explain the scavenging action of this compound, first proposed by Hardeland et al. [180]. In this hypothesis it is assumed that MLT radical cation—yielded by electron donation—is stabilized by resonance, thus having a rather long live. In turn this species reacts with O_2_^•−^ producing *N*^1^-acetyl-*N*^2^-formyl-5-methoxykynuramine (AFMK).

The evidence gathered so far on the AOC of MLT is so abundant, and compelling, that it has inspired the hypothesis that the primary function of MLT in living organisms is to protect them from OS [181]. MLT is capable of efficiently scavenging a wide variety of oxidants including ^•^OH [182], RO^•^ [183,184], CCl_3_OO^•^ [131], ^1^O_2_ [185,186], and ^•^NO [187,188]. Consequently, it reduces the molecular damage associated with FR generation in vivo [189]. MLT has also been found to inhibit lipid peroxidation [190,191,192,193,194]. In addition, the FR scavenging activity of MLT is shared by its metabolites, which allows that its AOC remains after MLT is metabolized. Such continuous protection is known as the antioxidant cascade [178,195,196], and makes MLT an exceptionally successful protector against OS, even at low concentrations.

It has been described that MLT exhibits protective effects against OS-related neurodegenerative disorders [197,198] and other OS-related diseases. For example, it was found to reduce the degenerative changes in experimental models of Alzheimer’s disease [199,200,201,202,203], while MLT administration to humans significantly slowed-down the progression of this disease [204,205]. MLT also has neuroprotective effect in Parkinson’s disease [206,207], and has been found to alleviate some of the secondary symptoms of cancer [208,209], and to be synergic with some radio- or chemo-therapies [210,211,212,213,214]. In addition, MLT reduces the toxic effects of some anti-cancer drugs like cisplatin and gossypol [182,215]. Moreover, it has even been determined that MLT may be effective in controlling metastatic breast cancer, both in vitro and in vivo, not only via inhibition of the proliferation of tumor cells but also through direct antagonism of a metastatic mechanism [216]. The antioxidant activity of MLT has been associated with its role in limiting ischemia-reperfusion injuries in the central nervous system (CNS) [217,218,219,220], heart [221], kidneys [222], liver [223,224,225], and lungs [226]. In addition, it has been hypothesized that melatonin may be synthesized in mitochondria [227], which would make MLT—and its metabolites—available for protecting mitochondria from OS [228].

On the other hand, MLT also increases the protective effects of glutathione, ascorbic acid and Trolox [229,230]. This was attributed to the capability of MLT to regenerate them by electron transfer processes. Repairing damaged biological targets is another pathway involved in secondary AOC. It has been found that MLT promotes the repair of oxidized DNA [179,231,232,233]. This is probably due to the MLT’s capability of transforming guanosine radical to guanosine by electron transfer [179]. As a secondary antioxidant, MLT has also been identified as efficient for counteracting the cytotoxic action of ^1^O_2_ [185] yielding AFMK [234].

MLT chelates aluminum, cadmium, lead, zinc, iron and copper [235] and drastically decreases the amounts of FR yielded by the interaction of Cu(II), Fe(II), Zn(II), Al(III) and Mn(II) with the β-amyloid peptide [236]. MLT also lowers the Cu(II)/H_2_O_2_-induced damage to proteins [237] and protects against copper-mediated lipid peroxidation [238], which led to the suggestion that the antioxidant and neuro-protective effects of melatonin may involve removing toxic metals from the CNS [238]. The protection of MLT against metal-catalyzed molecular damage was recently reviewed, and it was suggested that MLT may prevent the copper-induced FR generation in vivo by binding this metal [239]. Thus, according to the evidence gathered so far, it can be stated that MLT is an efficient multiple-function antioxidant.

### 3.3. Phenolic Compounds

Phenolic compounds are—arguably—the chemical family most widely investigated in the context of AOC. Their ability to protect against OS, and the associated health disorders has been profusely documented [46,48,240,241,242,243]. Like MLT, they are versatile antioxidants, able to deactivate diverse oxidants by various reaction mechanisms. Regarding their role as primary antioxidants, phenols have been found to scavenge FR mainly by HAT [244,245,246,247,248,249,250,251,252,253,254,255,256,257,258,259,260,261,262,263,264,265,266,267,268,269,270,271,272], PCET [273,274,275,276,277], and SPLET [278,279,280,281,282,283,284,285,286,287,288,289,290,291,292,293,294,295,296,297,298]; albeit, they can exert such protective action also by SEPT [299,300,301], RAF [302], SET-I [303,304] and SET-II [283,305]. As secondary antioxidants, phenolic compounds are capable of quenching ^1^O_2_ [306,307,308,309,310,311], repairing biomolecules [312,313,314,315] and chelating metals [316,317].

There is also abundant evidence on the beneficial health effects of phenolic compounds, which have been attributed to their antioxidant activity [48]. For example, they have been found to be effective in the prevention and therapy of skin disorders [318], to protect against cardiovascular [319,320,321,322] and neurodegenerative diseases [323,324], liver failure [325], and the toxic side effects of some pharmacological drugs [326]. In addition to their antioxidant related benefits, they have also shown anti-inflammatory [327] antimicrobial and antitumoral properties [50].

Phenolic compounds, particularly polyphenols are frequently present in the human diet. For example they are rather abundant in a wide variety of foods and beverages, including fruits, vegetables, wine, coffee and tea [328]. It has been reported that, after the intake of 10–100 mg of a particular phenolic compound, its maximum concentration in plasma rarely exceeds 1 mM [329]. However, it has also been assumed that the total phenol concentration in plasma is probably higher because of the presence of metabolites formed in body’s tissues or by the colonic microflora, which are still mostly unknown and not accounted for in the reported estimations [329]. At the same time, it seems that bioavailability can significantly change for different phenolic compounds, and that the most abundant phenols in our diet are not necessarily those with the best bioavailability profile [330].

## 4. Phenolic Melatonin-Related Compounds

**Figure 2:**
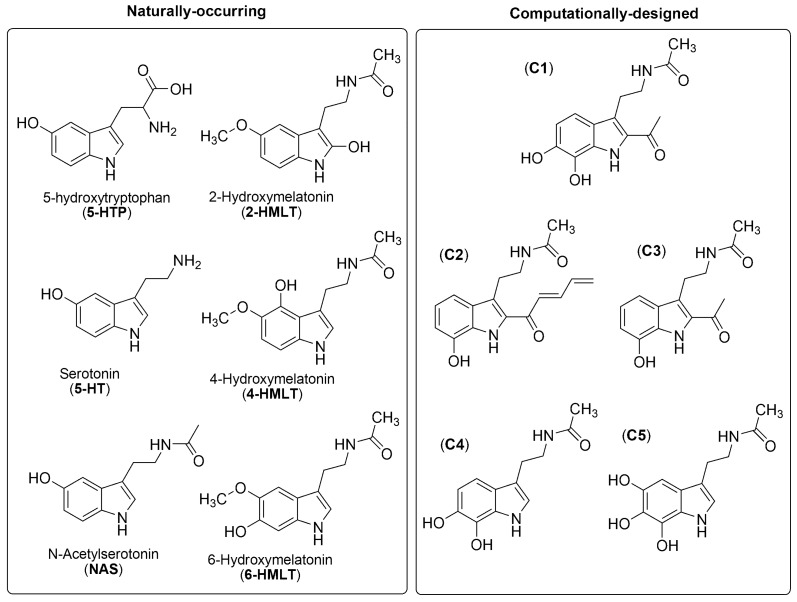
Phenolic melatonin-related compounds analyzed in this review.

Considering all the beneficial effects and the AOC of both MLT and phenolic compounds, it seems logical to assume that combining their structural features would lead to species with boosted or synergic activities. The phenolic MLT-related compounds analyzed in this review are shown in Figure 2.

**Figure 3:**
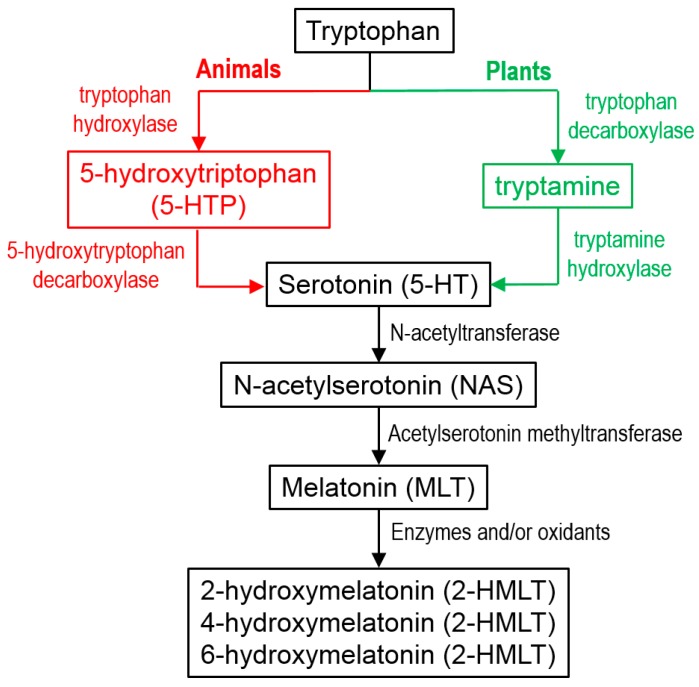
Schematic representation of the metabolic route connecting the naturally occurring phenolic melatonin-related compounds. Enzymes include cytochrome P450 (CP450), horseradish peroxidase (HRP), indoleamine 2,3-dioxygenase (IDO), eosinophil peroxidase (EPO), myeloperoxidase (MPO) and melatonin 2-hydroxylase (M2H). Oxidants include ROS and RNS.

Some of them are naturally-occurring molecules, while others were recently proposed as very promising antioxidants, based on computational-design strategies. The natural ones are involved in the tryptophan metabolic pathway in animals or plants (Figure 3). However, it is known that the metabolism of MLT is a highly complex process, involving both enzymatic and non-enzymatic (FR-induced) degradation [331]. The resulting products frequently overlap, making it difficult to identify which is the dominant degradation route, albeit under OS conditions the FR pathway is assumed to be the major one. In addition to what is shown in Figure 3, 2-hydroxymelatonin (2-HMLT) and 4-hydroxymelatonin (4-HMLT) are produced during the UV-induced metabolism of MLT [332]. 6-hydroxymelatonin (6-HMLT) is the primary hepatic metabolite of MLT in animals, while in plants 2-HMLT is the most abundant one [331,333]. This compound is also yielded by the oxidation of MLT by taurine chloramine [334].

The computationally-designed compounds shown in Figure 2 were chosen from a set of 19 melatonin analogues, intended to be better antioxidants than MLT, and to present both primary and secondary AOC. They were found to be among the best peroxyl radical scavengers identified so far, in aqueous solution, at physiological pH, and capable of downgrading ^•^OH production [53].

### 4.1. Location and Sources

The production of 5-hydroxytryptophan (5-HTP) and serotonin (5-HT) starts with dietary intake of L-tryptophan, which is present in a wide variety of foods including egg whites, chocolate, cod, dairy products, nuts and meats [335]. 5-HTP can also be taken as a dietary supplement, and it has been reported that it is well absorbed from consumed sources, with ~70% of the intake reaching the bloodstream [336]. In addition, the intestinal absorption of 5-HTP is not affected by the presence of other amino-acids, and it does not require a transport molecule. Accordingly, it may be taken with meals without decreasing it efficiency [337]. In addition, 5-HTP easily crosses the blood-brain barrier, thus it effectively contributes to increase the 5-HT synthesis in the CNS. The amount of endogenous 5-HTP available for this purpose depends on the availability of tryptophan, as well as on the activity of different enzymes; while the amount of 5-HTP that reaches the CNS is affected by how much 5-HTP is converted into 5-HT in the periphery [338].

5-HT is a ubiquitous molecule in nature. In plants it can be found in vegetables, fruits and nuts [339]. Some examples are cherries, coffee, tomatoes, Chinese cabbage, spinach, hot pepper, chicory, green onion, strawberry, lettuce, and rice [340,341,342,343]. In animals 5-HT is present in both vertebrates and invertebrates. In the particular case of humans, under normal conditions, 5-HT accounts for less than 2% of the L-tryptophan intake, which leads to a daily production of ~10 mg serotonin [339]. 5-HT is the neurotransmitter most widely distributed in the brain, albeit its amount in the CNS represents less than 5% of the whole body content [344]. It can be found in the pineal gland, serotonergic neurons, spinal cord and platelets; as well as in the liver, lungs, thyroid, bronchi, thymus and pancreas. However, the largest amount of 5-HT (about 80% of total content) is found in the gastrointestinal tract, particularly in the enterochromaffin cells [345], which are considered the site of synthesis and storage of 5-HT from tryptophan [346].

5-HT, NAS, 2-HMLT and 6-HMLT are present in cutaneous melatonin synthesis and metabolism with the latter being the main metabolite in epidermal cells [228]. On the other hand, while 5-HT is abundant in plants, *N*-acetylserotonin (NAS) is not [347,348,349,350]. However, it seems important to note that it is not only a precursor of MLT, but MLT can also be metabolized back into NAS [351]. Therefore, regardless of their relative abundance, it expected to find one of them wherever the other is present.

There is rather scarce information on whether 2-HMLT is enzymatically catabolized in vivo [196]. What most of the gathered evidence supports is that it is a product yielded from the reactions of MLT with chemical oxidants, such as the hypochlorous acid [352], oxoferryl hemoglobin [353], and ^•^OH [354]. The latter reaction also yields 4-HMLT. In addition, during the oxidation of cytochrome C by H_2_O_2_, in vitro, 2-HMLT is produced as an intermediate in the formation of AFMK [355]. It has also been observed that UV-B radiation-induced keratinocytes transform MLT into 2-HMLT [332]. This compound was found in rice seedlings, supporting its in vivo production from the MLT metabolism in plants [356]. On the other hand, 6-HMLT has been identified in the cerebral cortex, serum, heart, kidneys and liver of mice [205,357]. It has also been identified as the major MLT metabolite in the human skin [358,359]. Both, 2-HMLT and 4-HMLT were identified as products of the UV-induced metabolism of MLT in keratinocytes and cell-free systems [332].

The current information on the location and potential sources of 2-HMLT, 4-HMLT and 6-HMLT is less abundant than that concerning other phenolic MLT-related compounds. However, since they are produced by the interaction of MLT with oxidants—including ROS and RNS—it is expected that 2-HMLT, 4-HMLT and 6-HMLT are present in the same places as MLT, particularly under OS conditions. Since MLT is a ubiquitous molecule that can be found in plants and in many animal organs [357,359,360,361,362,363,364], its hydroxylated derivatives can be also formed in all of them. However, being as reactive towards oxidants as it will be demonstrated in following sections of this review, they are expected to have very short lives, which would make them hard to detect (particularly in excretion fluids, like urine).

### 4.2. Functions and Toxicity

Some of the species reviewed here are multifunctional molecules. In this section, their functions—other than AOC—are briefly described. It seems important to note than under OS conditions, just because of their AOC, the amounts of these compounds may be decreased affecting their other functions. This is particularly important for 5-HTP, 5-HT and NAS, since, apparently, the biological role of 2-HMLT, 4-HMLT and 6-HMLT is mainly to deactivate oxidants.

As mentioned before, 5-HTP is directly involved in the synthesis of 5-HT, while the latter and NAS are crucial to the MLT production. It has been shown that oral administration of 5-HTP can be effective in the treatment of depression [336,365]. It is assumed that 5-HTP supplementation normalizes the synthesis of 5-HT, a deficiency of which is believed to cause depression [366]. In addition to depression, 5-HTP has been found to be effective in the treatment of several conditions, including insomnia, fibromyalgia, cerebellar ataxia, binge eating and chronic headaches [337]. It has also been reported that 5-HTP supplementation inhibits leukocyte recruitment, serotonylation and allergic inflammation, which led to propose 5-HTP as a potential candidate in the treatment of allergy/asthma and the associated anxiety/depression symptoms [367]. 5-HTP was also found to be effective in reducing seizure-induced respiratory arrest, and was proposed as a possible therapy for preventing sudden unexpected death in epilepsy [368].

5-HT is also a multi-tasking molecule. It is involved in numerous physiological processes, such as peripheral and CNS neurotransmission, blood pressure regulation and smooth muscle contraction [339]. Probably its best known function is as a neurotransmitter–neuromodulator in the CNS. Its signaling pathways are involved in sensory processing, emotion regulation, cognitive control, autonomic control, and motor activity. Consequently, 5-HT modulates anxiety, fear, mood, stress, appetite, sleep, cognition, aggression and sexual behavior [344,369]. It is also a target of several physiological regulatory mechanisms and modulators such as gene transcription, psychotropic drugs, steroids and neurotrophic peptides [370]. In addition, it has been proposed that autism spectrum disorder, schizophrenia, bipolar disorder, impulsive behavior and attention deficit hyperactivity disorder, are all characterized by a dysfunction in the 5-HT pathway [371]. Deficient brain 5-HT synthesis, during development and adulthood, has been related to long-term neurodevelopmental disorders such as aggression, negative emotionality, and antisocial behavior [372]. Moreover, 5-HT has been identified to be concentrated in distinct brain regions collectively known as “the social-brain” [373,374].

In the periphery, 5-HT has been proposed to act as a pro-aggregator and vasoconstrictor when released from aggregating platelets, as an autocrine hormone when released from the enterochromaffin cells in the gut, pancreas and elsewhere; and as a neurotransmitter in the enteric plexuses of the gut [339]. In addition, 5-HT may be involved in the pathophysiology of hiccups based on its role in regulating the smooth muscles in the gastrointestinal tract to increase the tone and facilitate peristalsis, its vasoconstrictor properties, and its ability to dilate blood vessels of the heart and skeletal muscles [346]. 5-HT has also been identified as an endocrine hormone, a paracrine factor, or a growth factor; involved in mucosal growth/maintenance, gastrointestinal motility, intestinal inflammation, enteric neurogenesis, hepatic regeneration and osteogenesis [345].

At the same time, at elevated levels, 5-HT becomes toxic, leading to what is commonly referred to as the serotonin syndrome (SS). Its symptoms are numerous and affect the cardiovascular, gastrointestinal, autonomic, muscular, and central nervous systems [335]. Some examples are hypertension, disorientation, dizziness, flushing, hyperthermia, hyperreflexia, and myoclonus. In addition, elevated levels of 5-HT in blood—which are associated to disruption of the 5-HT/NAS/MLT pathway—have been identified as the most common marker in autism spectrum disorders [375].

To minimize the risk of SS it has been recommended not to administer 5-HTP in combination with serotonergic antidepressants [337,338]. There has been some cases of SS in patients concurrently taken L-tryptophan and fluoxetine, or switching from one serotonin reuptake inhibitor to another. On the contrary, there are no reports associating SS with the consumption of 5-HTP in monotherapies or combined with other medications. The most frequent adverse effects of 5-HTP identified so far involve the gastrointestinal tract, and include vomit, nausea and diarrhea; albeit insomnia, headache, and palpitations have also been observed [338]. While intravenous administration of 200–300 mg of 5-HTP can induce memory impairment, confusion and anxiety; these effects seldom appear with oral administration, especially at lower doses. For example, at dosages lower than 50 mg/kg/day no toxic effects have been found in connection with 5-HTP administration [338].

NAS, like MLT, functions as a signal molecule triggering plant defense responses [350,376] and growth [377]. In animals, NAS and 6-HMLT protect keratinocytes against UVB-induced OS and DNA damage [378]. They also exhibit membrane stabilizing activity in liver injury models [379]. NAS (and also MLT) improves membranes fluidity under OS conditions [380,381], which led to presume that they stabilize cellular membranes by preventing FR-induced lipid peroxidation [228,380,382]. In addition, the potential role of NAS, and melatonin, in the treatment of multiple sclerosis has been recently highlighted [383]. It has also been found that NAS can protect against acute hepatic ischemia-reperfusion [384].

### 4.3. Antioxidant Activity

The combined AOC of MLT and its metabolites may be responsible for the melatonin’s ability of deactivating several equivalents of oxidants. It is an intricate process that can be characterized as multifunctional [385] involving not only FR scavenging activity but also secondary AOC. For example their role as metal chelators may be highly beneficial for reducing the associated OS, since it would lead to the inhibition of ^•^OH [385]. It has been proposed that MLT and its metabolites may act in a “task-division” way, with some of them being particularly efficient as FR scavengers, and others mainly behaving as metal chelators [43]. Since scavenging FR and other oxidants result in a decrease of the associated damaging events, it would also imply an inhibition of protein oxidation, lipid peroxidation, mitochondrial damage and DNA destruction. Therefore, the antioxidant protection exerted by MLT, and related compounds, is expected to be efficient in maintaining a healthy redox status.

#### 4.3.1. 5-Hydroxytryptophan (5-HTP)

It has been shown that 5-HTP is efficient in inhibiting OS-induced damage. For example, its beneficial effects in inflammatory diseases have been attributed to its AOC [386]. 5-HTP also inhibits iron-induced lipid peroxidation processes [387] and prevents the oxidation of proteins and lipids, induced by iron-ascorbate mixtures [388]. These effects may arise from the ability of 5-HTP to chelate metal ions [389]. 5-HTP is also capable of suppressing UV-induced apoptosis in human monocytes [390], to inhibit the oxidative damage induced by *tert*-butylhydroperoxide (*t*-BuOOH) on human fibroblast [391], to preserve membranes fluidity under OS conditions [392] and to suppresses inflammation and collagen-induced arthritis by decreasing the production of pro-inflammatory mediators [393]. Based on in vitro investigations, it has been proposed that 5-HTP is more potent as an ^•^OH scavenger than MLT and ascorbic acid [394] and Trolox [395]. In addition 5-HTP has been described as efficient for scavenging α,α-diphenyl-β-picrylhydrazyl (DPPH) and OH radicals, and to cause about 95% inhibition of linoleic acid peroxidation [396]. 5-HTP also shows ^•^NO scavenging activity [397] and AOC protection on hyperglycemia-induced oxidative stress [398], albeit in both cases it was described as less efficient than MLT.

#### 4.3.2. Serotonin (5-HT)

It has been proposed that 5-HT protects membranes from lipid peroxidation through its FR scavenging activity, which is mainly exerted in the aqueous phase, or at the water-membrane interface [399]. Such protective activity has been held responsible for the attenuated secondary tissue damage in the CNS when serotonin is released at sites of brain damage or inflammation [400]. The antioxidant activity of 5-HT was also demonstrated by its suppressive effects on phagocytosis-associated, luminol-enhanced chemi-luminescence [401]. Its action was rationalized in terms of its reactions with ROS, found to be dose-dependent, and led to the proposal that 5-HT may modulate various aspects of cell-mediated defense reactions.

The in vitro antioxidant and FR scavenging activities of 5-HT have been evaluated using different methodologies and butylated hydroxyanisole (BHA), butylated hydroxytoluene (BHT), α-tocopherol and Trolox as reference compounds [402]. It was found that 5-HT is capable of fully inhibiting lipid peroxidation of a linoleic acid emulsion, being more efficient for that purpose than the references. 5-HT was also found to effectively scavenge DPPH, 2,2′-azino-bis(3-ethylbenzo-thiazoline-6-sulfonic acid) (ABTS^•+^), and hydrogen peroxide (H_2_O_2_) [402] as well as *N*,*N*-dimethyl-*p*-phenylenediamine (DMPD) radical [403]. The activity of 5-HT for the latter was found to be higher than that of MLT, which was attributed to its phenolic group. In addition, it was proposed that 5-HT, by scavenging peroxidase-derived ROS, may protect human natural-killer cells from oxidative damage at inflammatory sites [404].

The AOC of 5-HT on neuronal tissues have been examined by studying the oxidative damages of brain synaptosomal components induced by Fe(II) and ascorbate [405]. The oxidation processes were efficiently attenuated by 5-HT, which was also capable of significantly decreasing ^•^OH production and restoring the synaptosomal Ca(II) uptake, lowered by Fe(II) and ascorbate. In addition, it has been reported that 5-HT also exhibits ferric ion reducing power and ferrous ion chelating activities [402]. Accordingly, it seems that 5-HT plays protective roles against OS by directly scavenging FR, and also by sequestering metals and inhibiting FR production.

#### 4.3.3. *N*-Acetylserotonin (NAS)

Although NAS is both a precursor and a metabolite of MLT, its protective effects against OS seem to be independent from those of MLT [380,406]. In fact, it was hypothesized that since about 15% of melatonin is demethylated into NAS in vivo, some of the beneficial effects of MLT might be attributed to NAS. In addition, it has been proposed that the antioxidant effects of NAS against ROS, induced by *t*-butylated hydroperoxide and diamide, are higher than those of MLT [407].

It has been reported that NAS offers neuroprotection by inhibiting autophagy activation and mitochondrial death pathways [408] and to protect from H_2_O_2_-induced OS injuries [409]. NAS also defends against 6-hydroxydopamine-induced neurotoxicity [410]. Moreover, it has been suggested that the neuroprotective properties of NAS—and MLT—might mediate their cognition-enhancing effects [411]. NAS also exhibits antioxidant and anti-aging activities [412], as well as protective effects against β-amyloid-induced neurotoxicity [411] and light-induced degeneration of retinal photoreceptor cells [413]. There is also evidence on the NAS role in inhibiting H_2_O_2_-induced cell death and ROS production in hepatocytes cells [409]. NAS also reduces lipid peroxidation caused by iron, H_2_O_2_ and 2,2′-azobis (2-amidinopropane), also known as AAPH [380,406,414,415]. It was found that NAS administration to mice resulted in decreased lipid peroxidation and increased glutathione peroxidase in brain and kidney [412]. It was also found that NAS suppressed the glutamate-induced lipid peroxidation in retinal homogenates [416]. The effects of NAS in reducing lipid peroxidation is responsible for the protective role played by NAS in preserving optimal fluidity of the biological membranes [381].

NAS inhibits copper-induced lipids oxidation [417,418,419] and Cr(III)-induced DNA oxidative injuries [420]. It also has protective effects against Fe(II)-ascorbate dependent lipid peroxidation [421], iron-induced lipid peroxidation and lipids autoxidation [422]. It has been suggested that the antioxidant effects of NAS might reinforce its anti-aging, cognition-enhancing, antihypertensive, antidepressant, and antitumor effects [423]. It was also proposed that NAS—and its derivatives—might be useful in protecting against OS-related disorders such as cell death and mutagenesis as well as diseases like cancer, sepsis, post-ischemic trauma, and Parkinson’s and Alzheimer’s diseases. NAS was also suggested to be directly involved in the self-protective antioxidant system of the retina, as an efficient physiological FR scavenger within the photoreceptor cell [406].

#### 4.3.4. 6-Hydroxymelatonin (6-HMLT)

Not only 6-HMLT has been identified as an effective protector against OS-induced molecular damage, but also it has been proposed (together with NAS) as responsible for some—or much—of the AOC of MLT in vivo [228]. It was also suggested that since NAS and 6-HMLT are more hydrophilic than melatonin, their FR scavenging actions are exerted mainly in the aqueous phase, or at the water-lipid interface, while MLT positions itself within the lipid bilayer where it protects membrane proteins against FR attacks [228].

6-HMLT has been found to inhibit thiobarbituric acid-induced lipoperoxidation, which is attributed to ROS [424]. It was also suggested that the AOC of 6-HMLT is even larger than that of MLT in this context, which might be because of the presence of a hydroxyl group in the benzene ring, i.e., a phenol moiety. 6-HMLT was found to protect against cyanide-induced OS, which was attributed to its ability to downgrade KCN-induced O_2_^•−^ generation [425]. 6-HMLT is also capable of protecting rat brain homogenates against iron-induced lipid peroxidation, in vitro [426], and against quinolinic-acid-induced oxidative neurotoxicity in the rat hippocampus by efficiently scavenging ROS [427]. It has been demonstrated that 6-HMLT (but also MLT, 5-HT and 5-HTP) is capable to quenching ^1^O_2_ at rather fast rates [186]. This finding led to the proposal that 6-HMLT should present neuro-protective effects [427].

Regarding its secondary AOC, it has been reported that 6-HMLT efficiently protects against oxidative damage induced by UV irradiation [428]. Moreover, 6-HMLT was proposed as partially responsible for the potential benefits of incorporating MLT into sunscreens. In addition, there is evidence that this compound can reduce Fe(II)-induced lipid peroxidation and necrotic cell damage in the rat hippocampus in vivo [429]. Accordingly, it seems that 6-HMLT exerts both primary and secondary AOC, albeit it has been characterized as a better primary antioxidant [43]. On the contrary, it was suggested that, in the presence of metal ions, 6-HMLT might induce DNA damage via non-o-quinone type of redox cycle leading to carcinogenic effects [97]. Therefore, despite of the fact that most of the investigations reported so far—on the OS related effects of 6-HMLT—indicate that it is beneficial, further studies aiming to provide more information on this subject are still desirable.

To the best of our knowledge, there are no reports yet on the independent AOC of 2-HMLT and 4-HMLT. They are mainly identified as oxidation products of MLT. However, they are structurally similar to NAS and 6-HMLT, considering that they all are mono-phenolic derivatives of MLT. Accordingly, it might be expected that they may also have protective effects against OS by scavenging FR or by any of the chemical routes involved in secondary AOC. These two compounds, definitively deserve further investigations regarding their potential roles as antioxidants.

#### 4.3.5. Computationally-Designed Molecules

Albeit numerous synthetic MLT derivatives have been obtained in the last years, to our best knowledge none of them have a phenolic group. However, very recently a series of computationally-designed molecules presenting this feature were investigated [53]. For that purpose the Density Functional Theory (DFT) was used. The calculations were carried out with the 6-311+G(d,p) basis set and the continuum solvation model based on density (SMD) [430] using water as solvent. The M05-2X and M05 functionals [431] were used for geometry optimizations and frequency calculations for the systems without and with copper, respectively. Thermochemical and kinetic information on the AOC of the designed compounds was obtained and use to identify the most promising molecules.

The primary AOC was evaluated using the reactions with HOO^•^, and all the modeled compounds were found to be much better peroxyl radical scavengers than MLT. All the molecules shown in Figure 2 were predicted to react with HOO^•^ faster than NAS, and at rates similar to those of the reactions with 6-HMLT. This is relevant since NAS and 6-HMLT were previously identified as particularly efficient for that purpose [43]. Moreover, compounds C1, C2 and C3 were predicted to react with the target radical at diffusion-limited rates, which makes them excellent candidates to be efficient as primary antioxidants. In addition, it has been previously reported that phenolic compounds can be regenerated under physiological conditions. Such regeneration takes place in such a way that these compounds can scavenge several radical equivalents in the process, two per cycle (one HOO^•^ and one O_2_^•^^−^) [257,260,261,263,272,432,433,434]. Accordingly, the modeled compounds are expected to be capable of deactivating several FR equivalents.

The secondary AOC was evaluated as the Cu(II) chelation ability, and the inhibition of ^•^OH production. The idea was that while Cu(I) is required for producing ^•^OH, Cu(II) is the most abundant and stable oxidative state of copper. Therefore, chelating agents capable of decreasing the viability of Cu(II) reduction should be effective for preventing, or inhibiting, ^•^OH production, via the Fenton reaction, and the consequential OS. Compounds C1, C2 and C3 were identified as capable of turning off the Cu(II) reduction induced by O_2_^•−^ and Asc^−^, thus inhibiting the associated ^•^OH production. C1 and C2 were identified as the species with the best multifunctional AOC, and both fulfill the Lipinski’s [435] and Ghose’s [436] rules for orally active drugs. Thus, it is assumed that they will not present problems of bioavailability, poor permeation or absorption. However, C1 was the one proposed as the best prospect for possible application based on potential toxicity and synthetic accessibility estimations. It is expected that computationally-designed antioxidants with promising estimated properties would be actually synthesized in the near future, so their protective effects against OS-related injuries can be tested.

#### 4.3.6. Chemical Pathways

The relative importance of the different reaction mechanism that may be involved in the AOC of chemical compounds is probably one of the least explored aspects of this area of investigation. Regarding the compounds reviewed here, more investigations in the subject are still needed, albeit some advances had been made. Hydrogen transfer has been identified as a major pathway for the reactions of 5-HTP and 5-HT with DPPH [437], and the HOO^•^ scavenging activity of NAS, and 6-HMLT, in lipid media [43]. However, it is important to note that no investigations have been made on whether they are actually HAT or PCET reactions.

The RAF mechanism was proposed as relevant for the reactions of α-hydroxyethyl radicals with 5-HTP, 5-HT and MLT [438], while the chemical route referred here as SET-I has been identified as important for the primary AOC of 5-HTP and 5-HT [437,438,439]. On the other hand, the SPLET mechanism has been characterized as the thermodynamically preferred antioxidant mechanism in water for MLT and 60 *meta*- and *ortho*-substituted MLT derivatives [440]. It was also identified as viable for 5-HTP when scavenging a wide variety of free radicals [441] and for the reactions of NAS and 6-HMLT with HOO^•^ when they take place in aqueous solution, at physiological pH [442]. Under the same conditions, the SPLET mechanism was proposed as the main chemical route for the HOO^•^ scavenging activity of the computationally-designed molecules shown in Figure 2 [53].

Regarding secondary AOC, metal chelation has been proven to be important for MLT and related compounds [238,239,385,402]. It has been proposed that NAS and 6-HMLT are capable of turning off the ^•^OH production induced by copper-ascorbate mixtures and partially inhibiting the HWR [43]. Therefore, NAS and 6-HMLT are predicted to behave mainly as OIL type (ii). On the other hand, the computationally-designed phenolic MLT derivatives C1, C2 and C3 were characterized as multi-functional antioxidants; while C4 and C5 were designed to be primary antioxidants. For all of them, SPLET was identified as the main reaction mechanism involved in their primary AOC.

### 4.4. Comparisons with Other Antioxidants

Trends in AOC are crucial to design efficient strategies against OS, since they allow identifying the most efficient compounds for that purpose. However, it is a difficult task since the available data was not necessarily acquired using the same assay, or under the exact same conditions. For example, it has been reported that the AOC trends for the same set of antioxidants can be different in oil/water emulsion than in bulk corn oil, and also depending on the pH and the AOC assay [443,444]. Prior et al. [162] have called attention to the current lack of a universal AOC assay, and according to Frankel and Meyer “There is a great need to standardize antioxidant testing to minimize the present chaos in the methodologies used to evaluate antioxidants” [163].

Despite of all the challenges involved in making fair comparisons among antioxidants, and the rather scarce amount of information on the subject for phenolic MLT-related compounds, some trends have been established using both experimental and theoretical approaches. According to the experimental evidence, NAS was identified as more effective than MLT—but similar to Trolox—as a ROO^•^ scavenger [407] and for protecting against iron and lipopolysaccharide-induced lipid peroxidation [412]. The same trend was reported for lipid autoxidation and iron-induced lipid peroxidation [421,422], the copper-mediated oxidation of low density lipoproteins [417], and the Fe(II)-ascorbate induced lipid peroxidation in the bovine retina [421].

Regarding the other phenolic compounds reviewed here, 6-HMLT was found to be more efficient than MLT for inhibiting the lipo-peroxidation induced by thiobarbituric acid, which is attributed to the production of ROS [424]. 5-HTP was described as a more potent ^•^OH scavenger than MLT [394,445] and also for scavenging DMPD [129]. The free radical scavenging activity of 5-HT was found to exceed that of BHA, BHT, α-tocopherol and Trolox [130]. In addition, 5-HTP and NAS were characterized as better antioxidants than BHT in a triglyceride-system, albeit they were ineffective in a liposome-system, while MLT is unable of protecting polyunsaturated fatty acids (PUFAs) against lipid peroxidation in both cases [446]. These findings support the idea that evaluating AOC depends on the assay system.

On the other hand, the QM-ORSA protocol was designed to obtain kinetic data based on the antioxidant definition by Halliwell and coworkers [447,448], i.e., “any substance that when present at low concentrations compared to that of an oxidizable substrate would significantly delay or prevent oxidation of that substrate”. Therefore, for a molecule to be successful as an antioxidant, it should react faster than the species needing protection, which implies that rate constants (*k*) would be an optimum criterion for making AOC comparisons. The most abundant data obtained so far using QM-ORSA, correspond to the HOO^•^ scavenging activity of antioxidants. Based on the results obtained with QM-ORSA, NAS and 6-HMLT have been proposed as more efficient for scavenging ROO^•^ than MLT [43] which is in line with the experimental evidence. In addition, the computationally-designed MLT derivatives shown in Figure 2 were also predicted to be better peroxyl scavengers than the parent molecule [53].

**Figure 4:**
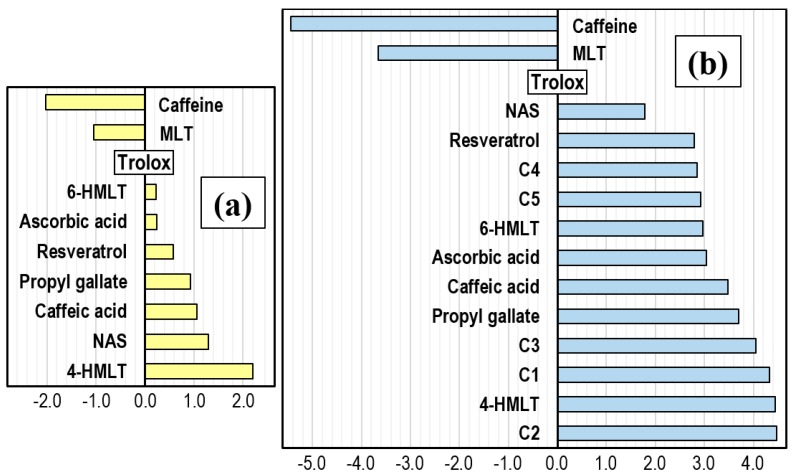
Relative rate constants (*k*) for the reactions with HOO^•^ (**a**) in lipid solution; (**b**) in aqueous solution, at pH = 7.4. The *x*-axis values correspond to log(*k*_i_/*k*_Trolox_). Data on Trolox, ascorbic acid, resveratrol, caffeine, propyl gallate, and caffeic acid was taken from references [120,173,263,272,292,456].

There are also experimentally-measured rate constants for the reactions between FR and the compounds reviewed here. They are reported in Table 3, where the label theoretical means that the *k* values were obtained with the QM-ORSA protocol. These values were estimated considering HOO^•^/O_2_^•−^ equilibrium to obtain rate constants in line with experimental measurements. HOO^•^ has a pKa equal to 4.8, which means that in aqueous solution, at pH = 7.4, its molar fraction is only 0.0025. Since the most abundant data correspond to the reactions with HOO^•^, a plot showing the trends in activity for scavenging this radical has been constructed to facilitate comparisons (Figure 4). In addition to the molecules of interest, the available data of other antioxidants, some of which are frequently used as reference, has also been included in this figure for comparison purposes.

According to the gathered kinetic data, MLT and its phenolic-related compounds all react at diffusion-limited rates with ^•^OH both in non-polar and polar solvents, i.e., they all are excellent ^•^OH scavengers. However, it is important to insist on the fact that this radical is highly reactive towards a wide variety of chemicals, despite the medium in which the reactions take place [457,458]. Thus, it is expected to react with almost any molecule in the vicinity of its formation site [115], which implies—arguably—that scavenging ^•^OH is not the best strategy to reduce OS, but to prevent its formation.

Despite the fact that the gathered kinetic data regarding ^•^NO_2_ and O_2_^•−^ is rather scarce, some trends can be established (Table 3). It seems that the pH has an important influence on the rate at which ^•^NO_2_ is scavenged by the analyzed compounds. For example, it has been reported that the rate constant of its reaction with 5-HTP increases from 9.0 × 10^5^ to 5.6 × 10^7^ M^−1^·s^−1^, as the pH goes from 5 to 9 [450]. This can be rationalized considering that 5-HTP is involved in acid-base equilibria, thus as the pH increases so do the molar fractions of the deprotonated species. Accordingly, it seems that the AOC of 5-HTP increases with its deprotonation degree. On the other hand, the *k* value for the MLT + ^•^NO_2_ reaction was estimated to be 3.7 × 10^6^ M^−1^·s^−1^, at pH = 7. Since the pKa of MLT is 12.3 [175], the fraction of its deprotonated species is negligible at neutral pH, and at any pH of physiological interest (3 ≤ pH ≤ 10). This means, that under such conditions, the reactivity of MLT towards ^•^NO_2_ should be rather independent of the pH. In addition, given the trend found for the reaction of 5-HTP with this radical at pHs equal to 5 and 9, it could be expected a rate constant in the order of 10^6^ M^−1^·s^−1^, at pH = 7. Accordingly, it seems fair to propose that MLT and 5-HTP should have similar ^•^NO_2_ scavenging activity at physiological pH (pH = 7.4). For the reactions with O_2_^•^^−^, the *k* values suggest that the reactivity trend is MLT < 5-HTP < 6-HMLT, but with only small differences in reactivity. In addition, since the *k* value for MLT (Table 3) was measured using water as solvent (presumably at acid pH), while for the other two the solvent was not specified, this trend needs further confirmation.

The data involving HOO^•^ is the most abundant, and probably the most adequate to make comparisons, since all the *k* values analyzed here were obtained in a similar way, i.e., using the QM-ORSA protocol, and at the same pH for the reactions in aqueous solution. The trends in primary AOC, based on the reactions with HOO^•^, is predicted to be 4-HMLT > NAS > caffeic acid > propyl gallate > resveratrol > ascorbic acid ≈ 6-HMLT > Trolox > MLT > caffeine, in non-polar (lipid) solution. Please note that the rate constants of C1-C5 were not included in the comparison because they have not been estimated yet in this media. In aqueous solution, at physiological pH, the primary AOC trend changes to C2 ≈ 4-HMLT ≈ C1 > C3 > propyl gallate > caffeic acid > ascorbic acid ≈ 6-HMLT > C5 > C4 ≈ resveratrol > NAS > Trolox > MLT > caffeine.

These trends strongly suggest that all the phenolic MLT-related compounds are better peroxyl radical scavengers than the parent molecule and Trolox, regardless of the polarity of the environment. This indicates that the presence of a phenolic moiety increases the FR scavenging activity within the MLT family. 4-HMLT was found to be in the top of the list in both cases, aqueous and lipid solution. The *k* values for 6-HMLT indicate that its primary AOC is similar to that of ascorbic acid but lower than that of propyl gallate and caffeic acid, also in both media. On the contrary, its relative activity with respect to resveratrol changes depending on the solvent’s polarity, i.e., it is lower in lipid and higher in water. Regarding the computationally-designed molecules, C2 and C1 seem to be the most promising ones as FR scavengers and as efficient as 4-HMLT, closely followed by C3. Moreover, they are among the best peroxyl radical scavengers reported so far. Their reactions with peroxyl radicals are predicted to be faster than those of any of the naturally occurring phenolic MLT-derivatives analyzed here, except 4-HMLT. Conversely, C4 and C5 are less efficient than 6-HMLT.

In addition, considering the above proposed trends, which identify 4-HMLT as the MLT metabolite with the highest primary AOC, a daring question arises: Is it actually 4-HMLT a minor metabolite of MLT (compared to 6-HMLT in animals and 2-HMLT in plants) or it is produced in amounts higher than previously thought but rapidly consumed because of its high reactivity towards FR? This probably deserves further investigations. In any case, what it seems to be clear is that 4-HMLT is a very efficient peroxyl radical scavenger, with higher activity than several widely recognized reference antioxidants.

Since the most abundant data gathered so far, concerning secondary AOC, involve metal chelation, it would be the one analyzed here. It has been found that MLT has a higher Fe(II) chelating ability than BHT, BHA and α-tocopherol [459], while NAS is more efficient than MLT for protecting against iron-induced lipid peroxidation [421,422]. NAS was also reported to be a better protector than MLT against copper-mediated oxidation of low density lipoproteins [417]. In addition, like their parent molecule, NAS and 6-HMLT were predicted to be capable of chelating Cu(II) [43,45,385]. However, although they were found to fully inhibit the ^•^OH production induced by Cu(II)-ascorbate mixtures, their inhibitory effects on the HWR were predicted to be only partial. Thus, the secondary AOC of MLT may be better than those of NAS and 6-HMLT.

Regarding the computationally-designed MLT phenolic derivatives, C1-C3 were identified as efficient for chelating Cu(II) [53]. Moreover, for Cu(II) chelates with them as ligands, the reactions with both O_2_^•−^ and Asc^−^ become endergonic and dramatically slower than those involving free copper. Accordingly, they were predicted as efficient for inhibiting ^•^OH production, under physiological conditions via HWR. Considering that they also are excellent for scavenging FR, these compounds were proposed as promising multifunctional antioxidants.

**Figure 5:**
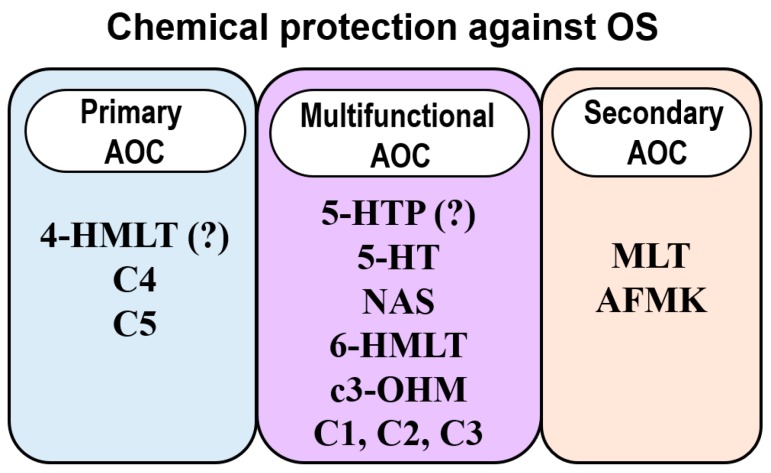
MLT, and related compounds, grouped according to their main AOC. A question mark next to the acronym of a compound means that further investigations are needed to confirm the proposed classification, thus its position can eventually change. AFMK = *N*^1^-acetyl-*N*^2^-formyl-5-methoxykynuramine, c3-OHM = cyclic 3-hydroxymelatonin.

Based on the previously analyzed data, a rough classification can be done regarding the AOC of the investigated compounds. It is provided in Figure 5, where MLT and some of its non-phenolic metabolites have been included for comparison purposes.

2-HMLT was not included in this figure because there is not enough quantitative data on the AOC of this compound. Hopefully, future investigations on this subject will provide more information on the AOC of 2-HMLT. In addition, the place of 5-HTP and 4-HMLT probably needs further confirmation. This has been indicated in the figure with a question mark next to their acronyms. It seems worthwhile to clarify that this classification was made based on current evidence, and considering the main kind of chemical protection exerted by the analyzed compounds against OS. However, it does not ruled out secondary AOC for a compound classified as primary, or vice versa. According to the gathered data, it seems that most of the phenolic MLT-related compounds are capable of counteracting OS as both, primary and secondary antioxidants, i.e., they present multi-functional AOC. This is a very desirable characteristic due to the wide variety of oxidants that are present in biological systems. This also means that they are not only able of scavenging FR, after they are produced, but also to inhibit their production under physiological conditions. Moreover, being as promising protectors against OS as they seem to be, they deserve further investigations that help to gain a deeper understanding on their chemical mechanisms of action.

## 5. Concluding Remarks

There is no doubt that OS represents a serious hazard to human health, thus molecules capable of offering protection against OS through chemical routes—in addition to the enzymatic pathways—are crucial to maintain a good health status. The phenolic and MLT families of compounds have both been identified as very efficient for that purpose. Accordingly, it is only logical to expect that phenolic MLT-related compounds are (at least) equally efficient as antioxidants. However, these compounds have been less investigated than phenols, MLT and its non-phenolic metabolites in the context of AOC.

The evidence gathered so far strongly indicates that MLT phenolic derivatives can act as both primary and secondary antioxidants. It also indicate that they may exert their protection against OS through diverse chemical routes including HAT, SPLET, metal chelation and inhibition of the ^•^OH production. All of the MLT-related compounds reviewed here seem to be better FR scavengers than MLT and Trolox, and most of them also surpass resveratrol, ascorbic acid, propyl gallate and caffeic acid.

On the other hand, OS and AOC are complex and manifold processes. They may involve multiple chemical species and reaction pathways, and may be influenced by several environmental factors such as the polarity of the media, the pH in aqueous solution, and the presence of other chemical species. There are many of these aspects that still deserve further investigations, particularly for the kind of compounds reviewed here. For example, very little is known regarding the potential AOC of 2-HMLT, or on the possible role of 4-HMLT as a secondary antioxidant. Albeit they were predicted to be efficient protectors against OS, the computationally-designed molecules have not been synthesized yet, so their AOC can be tested and the predictions might be confirmed or refuted.

Other challenges in this context are identifying the products yielded from the chemical AOC of phenolic MLT-related compounds (under physiological conditions), their relative abundance and their chemical fate. Further investigations are also desired regarding the possibility that they might be pro-oxidants under certain conditions, and their potential interactions with frequently used medical drugs.

## Figures and Tables

**Table 1:** Some of the most common oxidants in living organisms.

ROS		RNS		RSS	
Name	Acronym/Formulae	Name	Acronym/Formulae	Name	Acronym/Formulae
superoxide radical anion	O_2_^•−^	peroxynitrite	ONOO^−^	thiyl radicals	RS^•^
hydroxyl radical	^•^OH	nitric oxide	^•^NO	sulfenic acids	RSOH
alkoxyl radicals	RO^•^	nitrogen dioxide	^•^NO_2_	disulfide-S-oxides	RS(O)2SR
peroxyl radicals	ROO^•^				

**Table 2:** Most common reaction mechanisms involved in the primary AOC of chemical antioxidants.

Name	Acronym	Chemical Reaction *
Radical Adduct Formation	RAF	H*_n_*Atx + ^•^R → [H*_n_*Atx-R]^•^
Single Electron Transfer	SET	H*_n_*Atx + ^•^R → H*_n_*Atx^+•^ + R^−^ (I) H*_n_*Atx + ^•^R → H*_n_*Atx^−•^ + R^+^ (II)
Hydrogen Atom Transfer	HAT	H*_n_*Atx + ^•^R → H*_n_*_−1_Atx^•^ + HR
Proton Coupled Electron Transfer	PCET	H*_n_*Atx + ^•^R → H*_n_*_−1_Atx^•^ + HR
Sequential Proton Loss Electron Transfer	SPLET	H*_n_*Atx → H*_n_*_−1_Atx^−^ + H^+^ H*_n_*_−1_Atx^−^ + ^•^R → H*_n_*_−1_Atx^•^ + R^−^
Sequential Electron Proton Transfer	SEPT	H*_n_*Atx + ^•^R → H*_n_*_−1_Atx^•+^ + R^−^ H*_n_*_−1_Atx^•+^ → H*_n_*_−1_Atx^•^ + H^+^

* H*_n_*Atx = chemical antioxidant.

**Table 3:** Rate constants (*k*) of the reviewed compounds as primary antioxidants, i.e., as FR scavengers. MLT has been including for comparison purposes.

Antioxidant	FR	*k* (M^−1^·s^−1^)	Assay	Main Solvent	Reference
5-HTP	^•^OH	1.2 × 10^10^	Experimental	not specified	[449]
5-HTP	^•^NO_2_	9.0 × 10^5^	Experimental	water, pH = 5	[450]
5-HTP	^•^NO_2_	5.6 × 10^7^	Experimental	water, pH = 9	[450]
5-HTP	O_2_^•−^	1.2 × 10^4^	Experimental	not specified	[449]
5-HTP	DPPH^•^	~7 × 10^−1^	Experimental	methanol	[439]
NAS	DPPH^•^	~2 × 10^0^	Experimental	methanol	[439]
NAS	HOO^•^	6.70 × 10^4^	Theoretical	lipid	[43]
NAS	HOO^•^	1.17 × 10^6^	Theoretical	water, pH = 7.4	[53]
6-HMLT	^•^OH	1.1 × 10^10^	Experimental	water, pH = 7	[451]
6-HMLT	O_2_^•−^	2.7 × 10^4^	Experimental	not specified	[449]
6-HMLT	HOO^•^	5.81 × 10^3^	Theoretical	lipid	[43]
6-HMLT	HOO^•^	3.62 × 10^6^	Theoretical	water, pH = 7.4	[53]
4-HMLT	HOO^•^	5.48 × 10^5^	Theoretical	lipid	This work
4-HMLT	HOO^•^	6.21 × 10^6^	Theoretical	water, pH = 7.4	This work
C1	HOO^•^	4.79 × 10^6^	Theoretical	water, pH = 7.4	[53]
C2	HOO^•^	6.79 × 10^6^	Theoretical	water, pH = 7.4	[53]
C3	HOO^•^	2.54 × 10^6^	Theoretical	water, pH = 7.4	[53]
C4	HOO^•^	1.61 × 10^5^	Theoretical	water, pH = 7.4	[53]
C5	HOO^•^	1.89 × 10^5^	Theoretical	water, pH = 7.4	[53]
MLT	^•^OH	2.23 × 10^10^	Theoretical	benzene	[131]
MLT	^•^OH	2.57 × 10^10^	Experimental	water	^(a)^
MLT	^•^OOH	3.11 × 10^2^	Theoretical	benzene	[131]
MLT	^•^OOH	1.99 × 10^1^	Theoretical	water, pH = 7.4	[131]
MLT	O_2_^•−^	<1.0 × 10^4^	Experimental	water	[452]
MLT	^•^NO_2_	3.7 × 10^6^	Experimental	water, pH = 7	[175]

(a) Average from the values reported in references [131,175,451,452,453,454,455].
